# Effects of curl type on hair fracture patterns in non‐chemically treated hair

**DOI:** 10.1111/ics.70095

**Published:** 2026-11-02

**Authors:** Alison Robinson, Paul Cornwell, Ernesta Malinauskyte

**Affiliations:** ^1^ TRI Princeton Princeton New Jersey USA

**Keywords:** fatigue testing, fracture patterns, hair breakage

## Abstract

**Background:**

The problem of high breakage in textured hair has been investigated by analysing the fracture patterns on hairs broken during fatigue testing.

**Methods:**

Female panellists, aged 18–55, were recruited in New Jersey, in the United States and in Cape Town, South Africa. None had chemically treated their hair for at least 6 months. Panellists were split into three groups based on their natural curl type: wavy (Walker Type 2), curly (Walker Type 3) or coily (Walker Type 4). Single fibre fatigue testing was performed on a Diastron CYC801 (Dia‐stron Ltd., Andover, UK) fitted with four individual test cells. The instrument was housed in a bench‐top environmental chamber to regulate the humidity. Optical microscopy was used to analyse the fracture patterns in the hair from 16 US panellists and 16 South African panellists. Samples were analysed across Walker Types 2, 3 and 4. The main break patterns were put into seven categories.

**Results:**

The results show that male/female breaks and tear fractures predominate across all hair types. The incidence of smooth/clean breaks was very low. Data also show that Hair Type 4 has significantly more tear fractures and fewer male/female breaks than Hair Types 2 and 3 (*p* < 0.05, Tukey multiple comparison test). Hair Type 4 also has significantly more split fractures, although this was not a commonly seen fracture type.

**Conclusions:**

These data suggest that repeated stretching and untwisting of very curly, Type 4, hair in fatigue experiments often creates longitudinal cracks. Cracks that then usually progress to form long tear fractures. In contrast, the repeated stretching of straighter hair (Types 2 and 3) normally creates cuticle cracks perpendicular to the fibre axis, which eventually develop into male/female fractures.

## Graphical abstract

The problem of high breakage in textured hair was investigated by analysing the fracture patterns on hairs broken during fatigue testing. Data suggest that repeated stretching and untwisting of very curly, Type 4 hair in fatigue experiments often create longitudinal cracks that then progress to form long tear fractures.

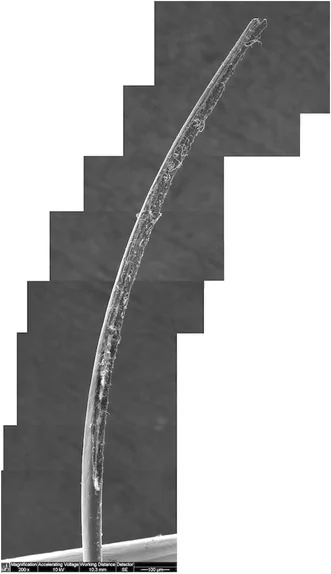

## Résumé

**Contexte:**

Le problème de la casse élevée des cheveux texturés a été étudié en analysant les profils de fracture des cheveux cassés lors d'essais de fatigue.

**Méthodes:**

Des panélistes de sexe féminin, âgées de 18 à 55 ans, ont été recrutées dans le New Jersey, aux États‐Unis, et au Cap, en Afrique du Sud. Aucune n'avait traité chimiquement ses cheveux pendant au moins six mois. Les panélistes ont été divisées en trois groupes en fonction de leur type de boucle naturelle: ondulée (Walker type 2) ou bouclée (Walker type 3) ou crépue (Walker type 4). Des tests de fatigue sur fibre unique ont été effectués sur un Diastron CYC801 (Diastron Ltd., Andover, Royaume‐Uni) équipé de quatre cellules de test individuelles. L'instrument a été placé dans une chambre environnementale de paillasse pour réguler l'humidité. La microscopie optique a été utilisée pour analyser les profils de fracture dans les cheveux de 16 panélistes américaines et 16 panélistes sud‐africaines. Les échantillons ont été analysés pour les Walker types 2, 3 et 4. Les principaux modes de rupture ont été classés en 7 catégories.

**Résultats:**

Les résultats montrent que les cassures mâle/femelle et les fractures par déchirure prédominent dans tous les types de cheveux. L'incidence des ruptures lisses/nettes était très faible. Les données montrent également que les cheveux de type 4 présentent significativement plus de fractures par déchirure et moins de cassures mâle/femelle que les cheveux de types 2 et 3 (*p* < 0,05, test de comparaisons multiples de Tukey). Les cheveux de type 4 présentent également significativement plus de fractures fendues, bien qu'il ne s'agisse pas d'un type de fracture fréquemment observé.

**Conclusions:**

Ces données suggèrent que les étirements répétés et le fait de détordre les cheveux très bouclés, de type 4, dans les expériences de fatigue, créent souvent des fissures longitudinales. Ces fissures évoluent ensuite en général pour former de longues fractures par déchirure. En revanche, l'étirement répété des cheveux plus raides (types 2 et 3) crée normalement des fissures de la cuticule perpendiculaires à l'axe de la fibre, qui finissent par se transformer en fractures mâle/femelle.

## INTRODUCTION

Single fibre fatigue experiments [1, 2] have shown an increased tendency for hair fatigue breakage as the degree of hair curl increases. Analysis of fatigue data showed that curlier fibres tended to break under fewer fatiguing cycles and exhibited a higher degree of premature failure.

Analysis of hair fracture patterns gives useful information about the mechanisms of fracture. Previous studies at TRI Princeton, for example, by Kamath et al. [3, 4], show that fracture patterns can reveal which structures in the hair are failing when hairs break. The predominant mode of fracture for textured hair, in conventional tensile experiments performed at high and low humidity, was the step fracture, suggesting the initiation of multiple cracks along the fibre axis [3, 4]. In the present study, we decided to look at the fracture patterns of fibres broken during fatigue testing, just to see if this gives us any new clues as to why textured hair breaks more easily.

In the present study, optical microscopy was used to take images of both ends of the hair fibre broken during fatigue measurements. The main break patterns were put into seven categories. These are built on the patterns originally described by Kamath et al., and are described in Figure 1.

**FIGURE 1:**
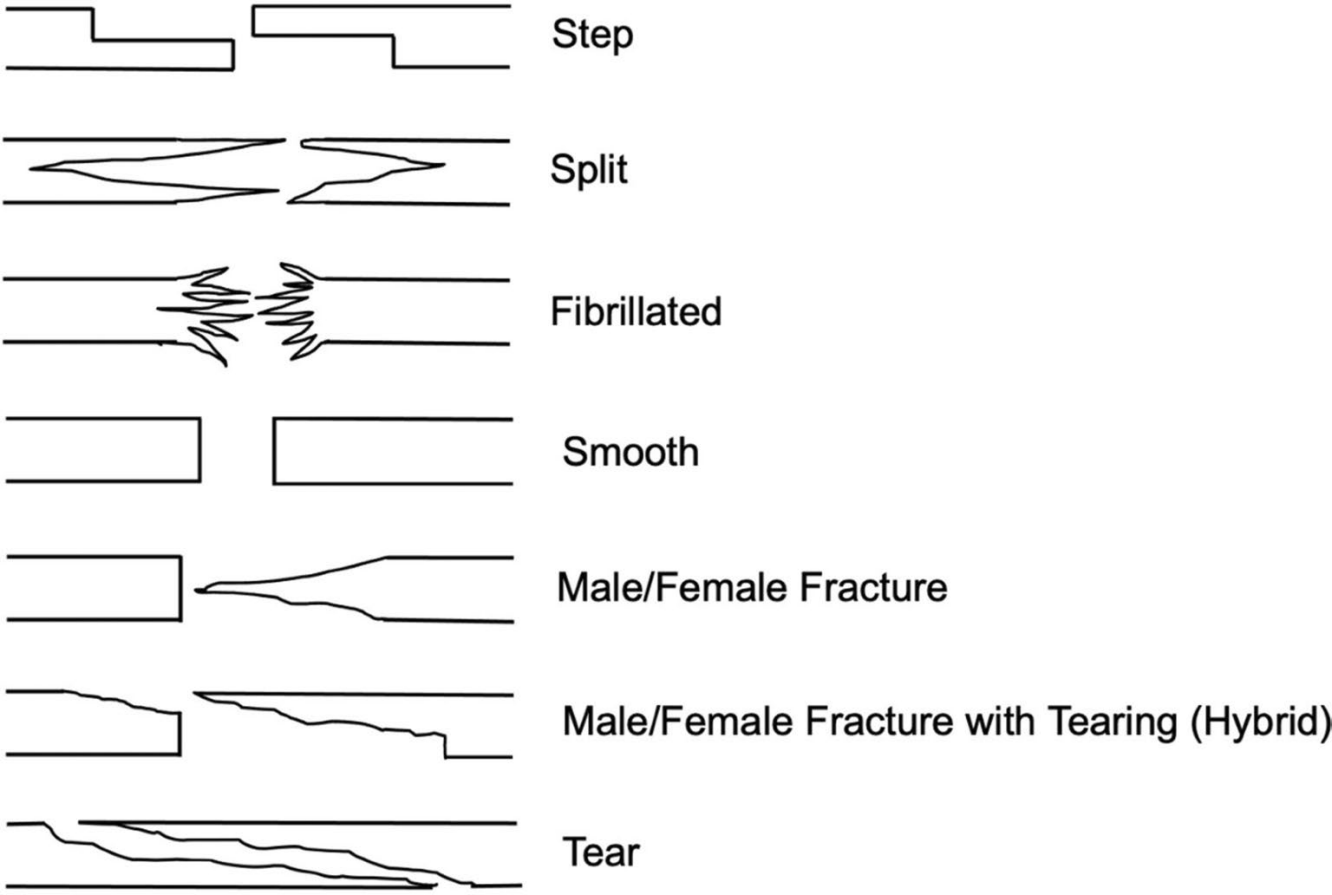
Principle types of fracture patterns.

## METHODS

### Clinical study

This study was performed in two locations, Piscataway, New Jersey (US) and Cape Town, South Africa (SA). Tests in the US were performed in July–August 2019 (US summer). Tests in SA were performed in November–December 2019 (SA late‐spring to summer).

This study recruited around 120 panellists in each country for a total of 243. Panellists were recruited based on their natural curl type: wavy (Walker Type 2a, 2b, 2c), curly (Walker Type 3a, 3b, 3c) and kinky (Walker Type 4a, 4b, 4c). In the US, 40, 39 and 39 panellists for Walker Hair Types 2, 3 and 4, respectively, were recruited. In SA, 40, 39 and 46 panellists for each curl type were recruited.

Subjects were all female, aged between 18 and 55 and had hair that was at least 6 cm in length or longer. All subjects self‐classified themselves as premenopausal or perimenopausal. A key exclusion criterion was that subjects must not have coloured or chemically treated their hair for at least 6 months prior to study start. All hair was, therefore, considered largely ‘natural’ and the scalp not exposed to chemical aggression.

Panellists were invited to visit the clinical centre on two occasions. A first baseline visit (Visit 1) and a second visit 1 week afterwards (Visit 2). At the baseline visit, the panellists' fit with the inclusion and exclusion criteria was assessed, and their hair types were assigned by a trained assessor. Hair samples were taken at this first visit.

### Hair sampling

Two hair bundles were collected as close to the scalp as possible. The samples were clipped from the crown of the head, in a location that was not easily noticed. The hair was at least 6 cm in length, and at least 100 hair fibres were taken from each site (total of 200 hair fibres per subject). Hair samples were secured at the root with adhesive tape and placed in Ziploc™ bags that were labelled with the subject number.

### Fatigue testing

Hair samples from a subset of 32 panellists were subjected to fatigue testing and for the analysis of fracture patterns. As the study focussed on textured hair, the majority of participants had Walker Type 4 hair. The distribution across Walker hair types was as follows: Type 2 (*n* = 5), Type 3 (*n* = 8) and Type 4 (*n* = 19). Subtype representation (a, b, c) was balanced within each type. Specifically, Type 2 comprised three 2a, one 2b and one 2c participants. Type 3 included three 3a, two 3b and three 3c participants. Type 4 consisted of six 4a, six 4b and seven 4c participants.

Fatigue testing revealed no observable differences in hair breakage between samples of equivalent curl types obtained from panellists in the US and SA. Furthermore, there is no literature evidence supporting the existence of such differences. Consequently, a balanced number of participants from both countries were included in the present study. For Walker Type 2, three panellists were from the US and two from SA. For Walker Type 3, four were from the US and four from SA. For Walker Type 4, nine were from the US and 10 from SA.

Single‐fibre fatigue testing was performed on a Diastron CYC801 (Dia‐stron Ltd., Andover, UK) equipped with four individual test heads. The instrument was housed in a bench‐top environmental chamber to allow for regulation of the relative humidity (RH). Experiments were performed in constant stress mode with a target stress of 115 MPa at 60 ± 2% RH and 22 ± 2°C. All fatiguing experiments were performed using a 40 mm/s extension rate.

Fatigue tests were performed, as is common practice, on 75 fibres from each panellist. Since fracture analysis was manual and very time consuming, not all the broken fibres could be analysed. Instead, a random subset of 45–50 pairs of broken fibres, with uniquely labelled crimps, was reserved for each panellist.

### Light microscopy

Broken fibres were stored after fatigue testing. Optical microscopy used a ×200 magnification. Images were examined by eye to determine the shape of the broken ends. Fibres were classified according to the naming system in Figure 1.

Approximately 45–50 hair samples were examined per panellist, meaning that over 1440 pairs of broken ends were classified in this study, all by the same scientist for consistency.

### Statistical analysis

Percentages of each break type were compared between hair types using ANOVA, followed by Tukey multiple comparison test (*p* < 0.05).

## RESULTS

Figure 2 shows the differences in break types across the different hair types. Data from the US and SA were combined. Statistical analysis of the results is shown in Table 1.

**FIGURE 2:**
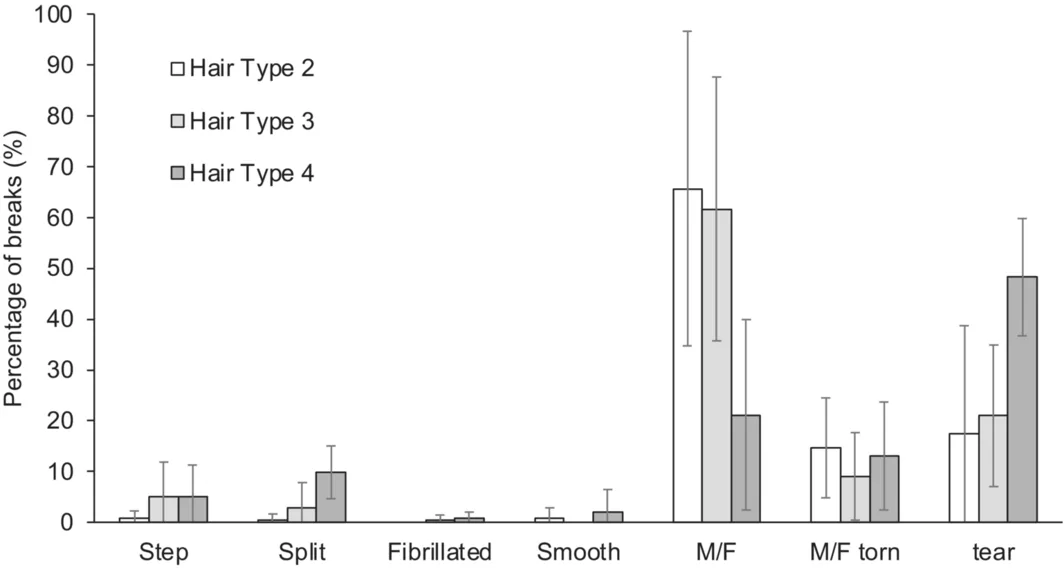
Summary histogram showing the effects of hair type on the fracture patterns seen after fatigue testing on dry hair (means ± standard deviation, *n* = 5 for Hair Type 2, *n* = 8 for Hair Type 3, and *n* = 19 for Hair Type 4).

**TABLE 1:** Summary table showing the effects of hair type on the fracture patterns seen after fatigue testing on dry hair.

Hair type	Number of breaks													
	Step		Split		Fibrillated		Smooth		M/F		M/F torn		Tear	
	Mean	Conf. groups	Mean	Conf. groups	Mean	Conf. groups	Mean	Conf. groups	Mean	Conf. groups	Mean	Conf. groups	Mean	Conf. groups
Hair Type 2	1	A	0	A	0	A	1	A	66	A	15	A	17	A
Hair Type 3	5	A	3	A	1	A	0	A	62	A	9	A	21	A
Hair Type 4	5	A	10	B	1	A	2	A	21	B	13	A	48	B

*Note*: Letters represent confluent groups identified by the multiple comparison test (Tukey, *p* < 0.05).

The results in Figure 2 and Table 1 show that, in fatigue testing experiments performed on dry hair, male/female breaks and tear fractures predominate across all hair types. The incidence of smooth/clean breaks was very low. Data also show that Hair Type 4 has significantly more tear fractures and fewer male/female breaks than Hair Types 2 and 3 (*p* < 0.05, Tukey multiple comparison test). Hair Type 4 also has significantly more split fractures, although this was not a commonly seen fracture type.

Figures 3 and 4 show SEM images of the two most frequently observed types of fracture patterns, the male/female breaks and tear fractures.

**FIGURE 3:**
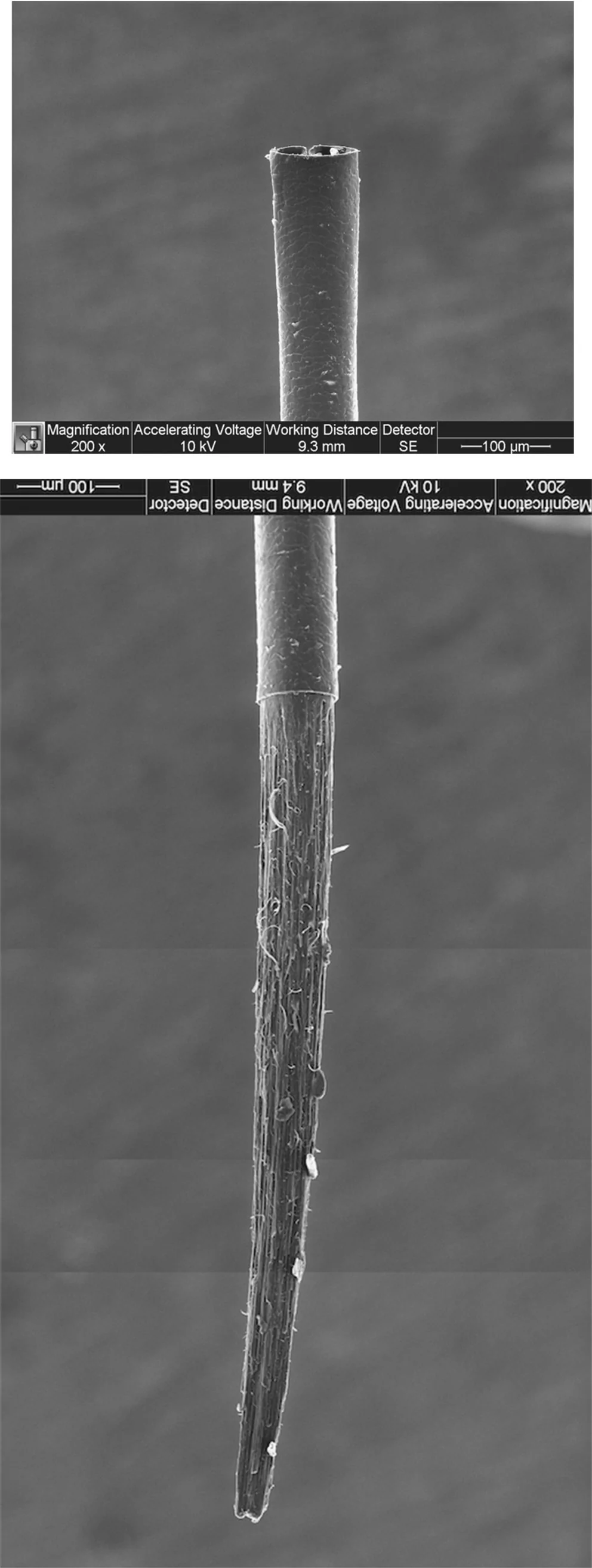
Each end of a typical male/female break (US, Walker Hair Type 3).

**FIGURE 4:**
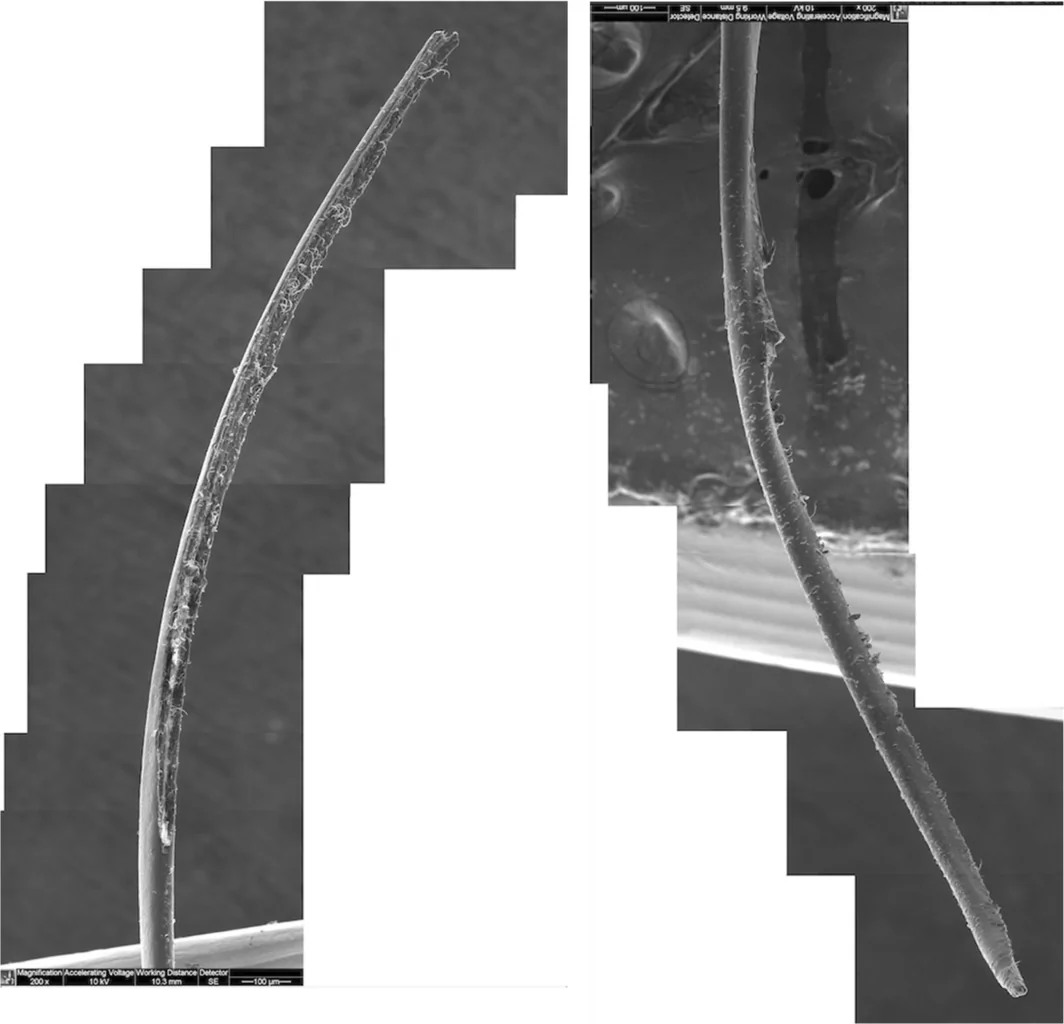
Each end of a typical tear fracture (US, Walker Hair Type 4).

## DISCUSSIONS AND CONCLUSIONS

These data suggest that in straighter hair types (Hair Types 2 and 3) fatigue stresses cause long fractures along the interface between the cortex and cuticle layers, leading to the creation of male/female break patterns. This is, perhaps, logical since the cuticle and the cortex have different tensile properties. The cuticle is significantly stiffer and possibly more brittle than the cortex [5].

One could postulate that cracks first form in the cuticle perpendicular to the fibre axis. Indeed, these cracks have previously been observed in fatigue experiments on straight hair [6]. These cracks might then completely encircle the fibre, transferring all the load to the cortex and the interface between the cuticle and cortex. Clearly, the interface between the cuticle and cortex is a weak‐point, since the cracks first run along this area, until, finally, the cortical cells fail, and the male/female break is formed.

In Hair Type 4, we saw significantly more tear fractures. Tear fractures probably start from the longitudinal fractures seen in unbroken hair samples of Type 4 hair. Figure 5 shows an example of this sort of crack from a previous study.

**FIGURE 5:**
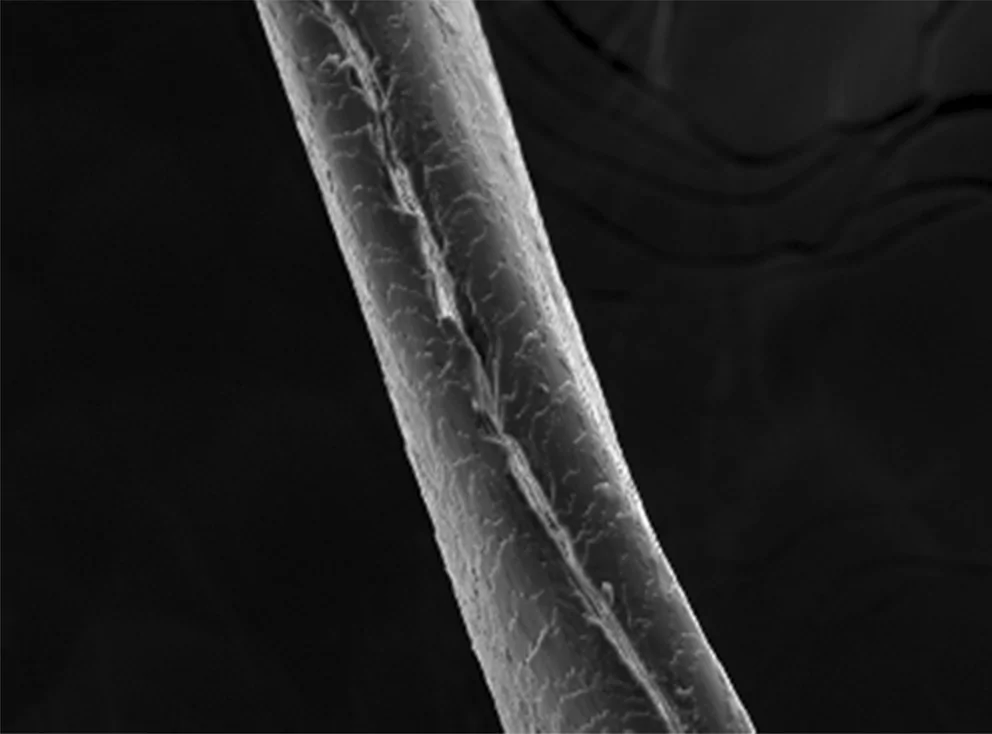
Longitudinal cracks in textured hair (without fatigue testing) [7].

It seems likely that stretching, untwisting and buckling very curly hair in fatigue experiments creates large torsional stresses on the fibre that initiate the formation of longitudinal cracks. Gamez‐Garcia suggests that stretching a coily hair fibre is similar to stretching a spring‐like helical structure and shows, through modelling evidence, that these torsional forces on the inside, versus the outside of the coils, cause intense stress points along the length of the fibres [8]. These stress points, he proposes, encourage cracks to form in the fibre along its length.

A similar mechanism has been proposed by Taylor et al. for the formation of split ends [9]. These authors show how repeatedly bending a damaged, straight hair fibre can eventually create longitudinal fractures and later splits. This process is thought to occur because the hair structure is relatively weak when stretched perpendicular to the main axis, which means that repeated bending of the hair can induce longitudinal fractures and split ends.

Camacho‐Bragada et al. used SEM and X‐ray tomographic microscopy to attempt to visualize breakage in Type 4 hair [10]. Their work was performed on single fibres of hair that underwent four strain cycles on a micro‐tensile tester. Data suggested that Type 4 broke in four stages: (i) cuticle sliding and lifting; (ii) failure of the cuticle–cortex interface; (iii) creation of cracks in between cortical cells and between the cortex and cuticle; and (iv) propagation of intercellular cracks towards the hair surface and the merging of cracks leading to breakage. This work was not able to connect these failure mechanisms to particular fracture patterns but is, nevertheless, consistent with the tear fractures observed in Type 4 hair in our study, which could have formed from cracks in between cortical cells.

In conclusion, these data suggest that repeated stretching, unwisting and buckling of very curly, Type 4 hair in fatigue experiments more frequently creates longitudinal cracks. Cracks that then progress to form long tear fractures. In contrast, the repeated stretching of straighter hair (Types 2 and 3) typically creates cuticle cracks perpendicular to the fibre axis, which eventually develop into male/female fractures.

Further work should investigate how these fracture patterns, observed in fatigue experiments, relate to those created by routine combing damage. Recent work at TRI suggests that grooming damage might create more fibrillated fractures [11], a type of fracture not often seen in the present study. It is possible that the rapid and intense forces created as hair is detangled by the brush might rip the hair apart in a different way to that in fatigue experiments.

Further work could also probe, in more depth, the exact location of the failure points in Type 4 hair at the micro‐level and at the macro‐level. In 1984, Kamath et al. [3], at TRI, suggested that fracture patterns seen in Type 4 hair were related to failures at the interfaces between cortical cells. Robbins et al. [12] also suggested that fractures in human hair often involve the rupture of intercellular bonds or the lipid cell membrane complex (CMC). Further work should investigate failure formation at the micro‐level and the exact role of the lipid CMC. For example, in‐depth analysis of the surfaces of fractured cortical cells could be used to investigate the relative quantities of exposed lipid CMC versus exposed internal cortical proteins.

Curly hair and wool fibres have different types of cortical cells in the inner portion of the curl, known as paracortical cells, and on the outside of the curl, known as paracortical cells [13, 14]. The different lengths and mechanical properties of these different cortical cells are related to hair curliness. Since the interfaces between hair components with different mechanical properties are common fracture points (e.g. the interface between cuticle and cortex), it is possible that the interface between ortho and paracortical cells could also be a weak‐point in Type 4 hair. Further work is currently taking place at TRI to investigate crack formation at this macro‐level.

## AUTHOR CONTRIBUTIONS

EM led the study. AR and PC conducted the study and analysed the data. PC authored the paper.

## FUNDING INFORMATION

Project sponsors in alphabetical order: Aveda and Bumble and Bumble (Estee Lauder), BASF, Croda, L'Oréal, Lubrizol, Procter & Gamble, Symrise, Syensqo (joined the project as Solvay), Unilever and TRI Princeton. One sponsor wishes to remain anonymous.

## CONFLICT OF INTEREST STATEMENT

The authors declare no conflicts of interest.

## Data Availability

The data that support the findings of this study are available from the corresponding author upon reasonable request.
